# Core Outcomes for Colorectal Cancer Surgery: A Consensus Study

**DOI:** 10.1371/journal.pmed.1002071

**Published:** 2016-08-09

**Authors:** Angus G. K. McNair, Robert N. Whistance, Rachael O. Forsythe, Rhiannon Macefield, Jonathan Rees, Anne M. Pullyblank, Kerry N. L. Avery, Sara T. Brookes, Michael G. Thomas, Paul A. Sylvester, Ann Russell, Alfred Oliver, Dion Morton, Robin Kennedy, David G. Jayne, Richard Huxtable, Roland Hackett, Susan J. Dutton, Mark G. Coleman, Mia Card, Julia Brown, Jane M. Blazeby

**Affiliations:** 1 Centre for Surgical Research, School of Social and Community Medicine, University of Bristol, Bristol, United Kingdom; 2 Severn School of Surgery, Bristol, United Kingdom; 3 Division of Surgery Head and Neck, University Hospitals Bristol NHS Foundation Trust, Bristol, United Kingdom; 4 Department of General Surgery, North Bristol NHS Trust, Bristol, United Kingdom; 5 Colorectal Consumer Liaison Group, National Cancer Research Institute, London, United Kingdom; 6 Academic Department of Surgery, University of Birmingham, Birmingham, United Kingdom; 7 Department of Surgery, St Mark’s Hospital and Academic Institute, Harrow, United Kingdom; 8 Academic Surgical Unit, St James’ University Hospital NHS Trust, Leeds, United Kingdom; 9 Centre for Ethics in Medicine, University of Bristol, Bristol, United Kingdom; 10 Colorectal Site Specific Group, Somerset, Wiltshire, Avon & Gloucestershire, South West Cancer Network, Bristol, United Kingdom; 11 Centre for Statistics in Medicine and Oxford Clinical Trials Research Unit, Nuffield Department of Orthopaedics, Rheumatology and Musculoskeletal Sciences, University of Oxford, Oxford, United Kingdom; 12 Department of Colorectal Surgery, Plymouth Hospitals NHS Trust, Plymouth, United Kingdom; 13 Clinical Trials Research Unit, University of Leeds, Leeds, United Kingdom

## Abstract

**Background:**

Colorectal cancer (CRC) is a major cause of worldwide morbidity and mortality. Surgical treatment is common, and there is a great need to improve the delivery of such care. The gold standard for evaluating surgery is within well-designed randomized controlled trials (RCTs); however, the impact of RCTs is diminished by a lack of coordinated outcome measurement and reporting. A solution to these issues is to develop an agreed standard “core” set of outcomes to be measured in all trials to facilitate cross-study comparisons, meta-analysis, and minimize outcome reporting bias. This study defines a core outcome set for CRC surgery.

**Methods and Findings:**

The scope of this COS includes clinical effectiveness trials of surgical interventions for colorectal cancer. Excluded were nonsurgical oncological interventions. Potential outcomes of importance to patients and professionals were identified through systematic literature reviews and patient interviews. All outcomes were transcribed verbatim and categorized into domains by two independent researchers. This informed a questionnaire survey that asked stakeholders (patients and professionals) from United Kingdom CRC centers to rate the importance of each domain. Respondents were resurveyed following group feedback (Delphi methods). Outcomes rated as less important were discarded after each survey round according to predefined criteria, and remaining outcomes were considered at three consensus meetings; two involving international professionals and a separate one with patients. A modified nominal group technique was used to gain the final consensus. Data sources identified 1,216 outcomes of CRC surgery that informed a 91 domain questionnaire. First round questionnaires were returned from 63 out of 81 (78%) centers, including 90 professionals, and 97 out of 267 (35%) patients. Second round response rates were high for all stakeholders (>80%). Analysis of responses lead to 45 and 23 outcome domains being retained after the first and second surveys, respectively. Consensus meetings generated agreement on a 12 domain COS. This constituted five perioperative outcome domains (including anastomotic leak), four quality of life outcome domains (including fecal urgency and incontinence), and three oncological outcome domains (including long-term survival).

**Conclusion:**

This study used robust consensus methodology to develop a core outcome set for use in colorectal cancer surgical trials. It is now necessary to validate the use of this set in research practice.

## Abstract 2

Angus McNair and colleagues describe 12 outcome domains that form a core outcome set for colorectal surgery research.

## Background

Randomized controlled trials (RCTs) represent the gold standard in evaluating health care interventions. They aim to produce high quality evidence that can be used to inform clinical care; however, the clinical impact of RCTs is diminished by of a lack of coordination of outcome measurement and reporting. Indeed, multiple systematic reviews throughout many different branches of medicine have been consistent in demonstrating the large number and heterogeneity of outcome reporting in trials and other research studies [1–4]. This has the effect of making clinically relevant comparisons between trials and pooling of results in meta-analyses difficult. Furthermore, multiplicity of outcome measurement can lead to the selective reporting of significant findings in the form of outcome reporting bias [5].

A proposed solution to these issues is to develop and use “core outcome sets” (COSs). A COS is a minimum set of outcomes that key stakeholders agree to be measured in all trials in a particular field [6]. This approach allows a consistent set of outcomes to be measured and has the potential to improve the efficiency with which research can answer clinical questions. The benefits of COSs have now been embraced internationally by funding bodies [6], regulatory bodies [7,8], and journal editors [9], all of which recommend their use where available. As a result, the development of COSs is increasingly common. The COMET (Core Outcome Measures in Effectiveness Trials) initiative has recorded nearly 600 published or ongoing studies into COSs, and many have now been developed in diverse clinical areas including rheumatology [10], pediatrics [11], and obstetrics [12]. There is, however, no established COS for colorectal cancer (CRC) surgery.

This is now urgently needed, because CRC surgery is undergoing a period of intense innovation. CRC is a major cause of worldwide morbidity and mortality, representing the third most common cancer and fourth most common cause of cancer death [13]. Surgery is a fundamental method for both curative and palliative treatment of this disease, and there is therefore a great need to improve the delivery of such care [14]. The last decade has seen several RCTs of laparoscopic techniques, all of which have measured different outcomes and thus suffer from the weaknesses described above [15,16]. The future will include evaluations of robotic surgery, transanal resection of the rectum, and organ-preserving rectal surgery, all of which have the potential to improve the care of many patients with CRC, provided they are evaluated in a robust and efficient manner. The aim of this study is therefore to define a COS for use in trials and other studies in CRC surgery, agreed upon by patients and CRC professionals.

## Methods

The scope of this COS includes clinical effectiveness trials (rather than trials of treatment efficacy) of all surgical interventions for cancer of the colon and rectum. Excluded were oncological interventions. The COS defines which outcomes are recommended, but does not specify how they should be measured. The COS could also be used in audit and nonrandomized studies of CRC.

The development of the COS was conducted in three phases according to COMET guidelines [6]. In Phase 1, a long list of outcomes that could be measured in CRC trials was identified, and outcomes were categorized into domains. In Phase 2, domains were operationalized into a questionnaire that was used to survey stakeholders’ views on the importance of each domain using Delphi methods. In Phase 3, consensus meetings with patients and surgeons were used to finalize the core set. Appropriate ethics regulatory approval was granted (National Research Ethics Service number 10/H0102/82).

### Phase 1: Domain Generation

Outcomes of CRC surgery were identified from three sources; i) systematic review of clinical and patient-reported outcome literature [17,18]; ii) interviews with patients; and iii) analysis of written patient information leaflets used for colorectal surgery in hospitals in the United Kingdom. Duplicates were removed, and a long list of outcomes was created. Similar outcomes were categorized into domains by two members of the study team. Patient-reported outcomes were grouped into domains (e.g., ability to walk and activity levels were grouped within the physical function domain) and verified by two researchers and a patient representative [19]. Items from patient information leaflets were independently categorized by two surgeons. Discrepancies were resolved through discussion with the study lead. Overlapping domains between data sources were condensed, producing a final list of domains.

The final domains were operationalized into questionnaire items using lay language with the medical terminology included in parentheses. The questionnaire was piloted by patients for face validity, understanding, and acceptability and modified as a result of this feedback.

### Phase 2: Delphi Consensus Methods

The questionnaire developed in Phase 1 was sent to stakeholders including CRC surgeons, clinical nurse specialists, and patients who had undergone surgery for CRC (Round 1). Patients were considered to be essential stakeholders, as they are the recipients of treatment, and surgeons and clinical nurses have an in-depth understanding of the potential impact of surgery. Oncologists were excluded as chemo/radiotherapy was outside the scope of this COS. Surgeons and nurses were identified from United Kingdom (UK) National Health Service hospital trusts that routinely performed surgical resection of CRC and participated in the UK National Bowel Cancer Audit. Nonprobabilistic purposive sampling was conducted to ensure center variation based upon geographical region (Northern England, the Midlands, Southwest and Southeast England, and Wales) and caseload volume per annum as determined by number of major resections in 2012. Patients were recruited from University Hospitals Bristol NHS Foundation Trust, North Bristol NHS Trust, and Plymouth Hospitals NHS trust in the UK. Participants were approached by post and were sent the questionnaire with a stamp-addressed envelope for return. One reminder was sent if there was no response after four weeks. Nonprobabilistic purposive sampling was conducted to ensure representation based on age, sex, and cancer site (rectum, left colon, right colon). Demographic data was collected including area of deprivation, marital status, employment status, and educational level. Deprivation was defined by the UK Office of National Statistics Index of Multiple Deprivation at lower layer Super Output Area level for the individual. This is a combined measure of income, employment, health and disability, education, barriers to public services, crime, and living environment. Educational level was defined as up to basic education (to the age of 16 or completion of the UK General Certificate of Secondary Education or equivalent), further education (subsequent qualifications to the age of 18 but not degree level), undergraduate, and postgraduate education.

Questionnaires asked participants to rate the importance of domains on a nine-point Likert scale, where 1 was a “not essential” and 9 an “absolutely essential” outcome. Returned first round questionnaires were analyzed, and any outcomes considered least essential were discarded. In Round 2, participants were provided with feedback from Round 1 in the form of their previous score for each domain and a mean score from their stakeholder group. Participants were then asked to rescore each domain on the nine-point Likert scale, and the results were used to determine which domains should be retained and presented in the consensus meetings. Participants that did not respond to the first questionnaire were ineligible for Round 2 because of the necessity to receive their own feedback. Responses from Round 1 were accepted until the Round 2 questionnaire was distributed. Round 2 responses were accepted until the respective stakeholder consensus meeting.

### Phase 3: Face-to-Face Consensus Meetings

Three consensus meetings were held; two with health professionals and a third with patients and caregivers. The first professional consensus meeting was held at the Tripartite Colorectal Meeting (the combined meeting of the Association of Coloproctology of Great Britain and Ireland, the American Society of Colon and Rectal Surgeons, the Royal Society of Medicine, Royal Australasian College of Surgeons, Colorectal Surgical Society of Australia and New Zealand, and the European Society of Coloproctology) in Birmingham in 2014. Ongoing discussion prevented the completion of the consensus meeting within the allotted time, and a second was hosted by the European Society of Coloproctology meeting in Barcelona in 2014. Meetings were open to all members of international societies and, in addition, all participants of the Delphi process were invited to attend. Participants were asked to declare their country of residence. The patient and caregiver meeting was held in Bristol in 2013. Attendees at this meeting were all from the UK and had completed the questionnaire surveys and responded to an invitation to attend a consensus meeting.

The retained outcomes from the second survey were presented at the meetings, and participants were asked to anonymously rate their importance. Anonymized voting took place to ask participants to vote each outcome as either “In” or “Out” using electronic keypads. Histograms and descriptive statistics were created live for each outcome during the meeting and displayed to the participants. Where the similar number of participants voted “In” or ‘“Out,” issues were explored by discussion to determine the nature of the polarized response within the stakeholder groups. Dissenting views were actively sought and considered before voting was completed.

### Sample Size

There are no agreed methods to set the sample size for Delphi surveys or consensus meetings, and there is no requirement for a statistically representative sample [20]. Therefore, an opportunistic approach was used with the aim of obtaining approximately 100 respondents for both the professional and patient stakeholder groups for the survey and a smaller group in which discussion could take place in the consensus meetings.

### Data Analyses

After Round 1 of the survey, outcomes were categorized as “essential” and retained for Round 2 if they were rated between 7 and 9 by over 50% of respondents and between 1 and 3 by less than 15%. Outcomes not meeting these criteria for either patients or professionals were discarded. Mean scores were calculated for each retained outcome to form the feedback for Round 2. Round 2 responses were analyzed with stricter cut-off criteria, retaining outcomes rated between 7 and 9 by over 70% of respondents, and between 1 and 3 by less than 15%. There are no agreed methods for selecting cut-off criteria within Delphi studies and, therefore, the criteria were chosen after discussion within the writing group and collaborators within the COMET initiative.

The outcomes retained after Round 2 were considered in Phase 3 consensus meetings. During the meeting, each outcome was discussed, and voting took place that asked attendees to vote outcomes as “In,” “Out,” or during the patient meeting, “Unsure.” The “Unsure” category was included in the patient consensus meeting to ensure that participants understood the question. Voting was undertaken using electronic keypads to ensure anonymity, and no data were collected on participants who changed votes. The unsure items were rediscussed with further voting and discussion. All items retained from the patient and professional meetings were included in the final core set. There is no accepted definition of consensus in the literature. The overall approach in this study was to be inclusive so that outcomes of importance to participants were not inappropriately excluded from the COS. Therefore, outcome domains were only excluded if voted “In” by less than 33% of participants. There were deviations from this analysis. In the first professional consensus, meeting a more conservative approach was taken, because there was insufficient time for discussion. Domains were only excluded if voted “In” by less than 25% of participants. In addition, if consensus was not reached after two rounds of professional voting, a majority rules approach was taken.

## Results

### Phase 1: Domain Generation

**Fig 1:**
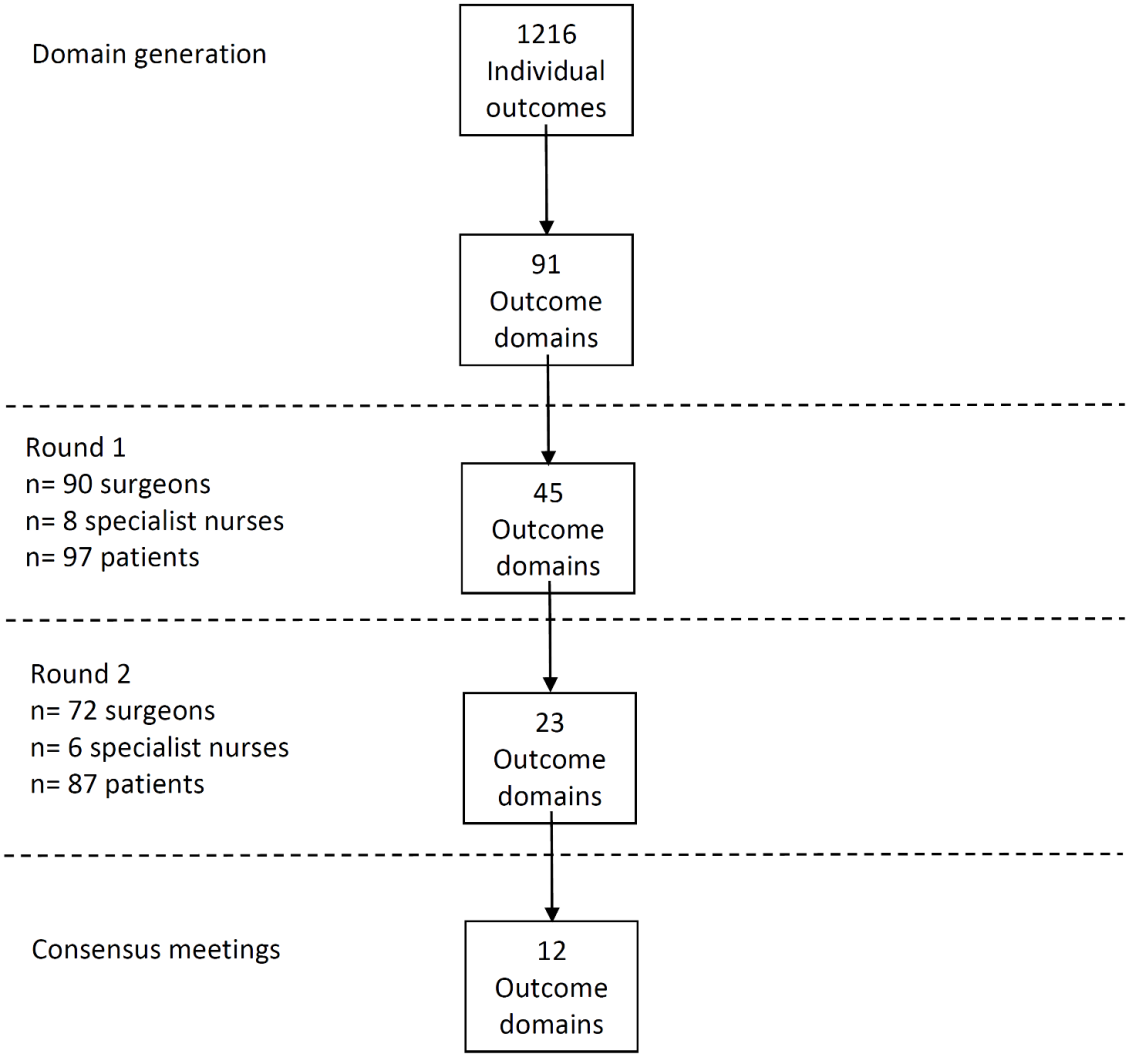
Flow diagram of Delphi process.

Review of all data sources identified 1,216 outcomes of CRC surgery that were grouped into 91 domains. The domains included outcomes about survival, recurrence, postoperative complications, and long-term quality of life. A summary of results is presented in Fig 1.

### Phase 2: Delphi Process

A total of 81 CRC centers were sampled, of which 63 (78%) responded, including 90 surgeons and 8 clinical nurse specialists (Table 1). The centers represented all geographical regions of England and Wales, and caseloads averaged 117 major resections per year (range 38 to 275). Patient response rate was 97 out of 267 invited (36%). The patients’ age range was wide (29 to 87), sex ratio fairly equal (41 female, 42%), and similar numbers of patients had rectal (33, 35%), left (34, 35%), and right (30, 29%) colonic tumors. Many patients lived in areas of low deprivation, but there was an even distribution of basic and higher educational level. Health professionals rated short-term technical outcomes of greatest importance in Round 1 including anastomotic leak, adequacy of resection margins, and perioperative mortality (Table 2). Although these issues were also rated as important to patients, patients gave a major priority to longer term outcomes such as survival, distant recurrence, and impact on longer term quality of life. A total of 45 domains met the criteria to be retained for Round 2 (S1 Table).

**Table 1:** Participant characteristics.

	Clinical centers	
	Responders (63)	Nonresponders (18)
Region (%)		
Northern England	14 (22)	5 (28)
Midland	8 (13)	0
Southeast England	22 (35)	10 (55)
Southwest England	9 (14)	0
Wales	10 (16)	3 (17)
Mean number of major colorectal resections (range)^a^	117 (38 to 275)	90 (29 to 210)
	Patients	
	Responders (*n* = 97)	Nonresponders (*n* = 170)
Mean age (range)	64 (29 to 87)	68 (29 to 88)
Female (%)	41 (42)	95 (56)
Cancer site (%)		
Rectum/anus	33 (35)	55 (32)
Left colon	34 (36)	46 (27)
Right colon	30 (29)	60 (36)
Unknown		9 (5)
IMD quintile (%)^b^		
1	5 (5)	27 (16)
2	13 (13)	38 (23)
3	20 (21)	24 (14)
4	20 (21)	41 (24)
5	39 (40)	23 (23)
Educational level (%)		
Basic	30 (32)	
Higher	34 (35)	
Undergraduate	16 (16)	
Postgraduate	6 (6)	
Not disclosed	11 (11)	
Marital status (%)		
Single/divorced	17 (18)	
Married/cohabiting	73 (75)	
Widowed	7 (7)	
Employment status (%)		
Employed	16 (17)	
Retired	58 (60)	
Seeking work	1 (1)	
Not working voluntarily	5 (5)	
Sickness leave	5 (5)	
Other	12 (12)	
Length of hospital stay (%)		
<2 weeks	80 (83)	
2–3 weeks	10 (10)	
3–4 weeks	3 (3)	
>4 weeks	4 (4)	

^a^Number of major cancer resections are defined by the UK National Bowel Cancer Audit 2012.

^b^IMD: Index of Multiple Deprivation as defined by the UK Office of National Statistics at lower layer Super Output Area level for the individual. Lower quintile equates to higher deprivation.

**Table 2:** Top ten highest scored outcome domains after Round 1, by stakeholder group.

Outcome domain	n (%) patients rating domain highly important^a^	Outcome domain	n (%) professionals rating domain highly important^a^
	*N* = 97		*N* = 98
Resection margins	88 (91)	Anastomotic leak	96 (99)
Stoma rate	84 (87)	Resection margins	93 (96)
Distant recurrence	81 (83)	Operative mortality	89 (92)
Recurrence	80 (82)	Conversion to open operation	88 (91)
Local recurrence	80 (82)	Distant recurrence	87 (90)
Nonprogression	80 (82)	Re-operation	87 (90)
Disease free interval	79 (81)	Local recurrence	86 (89)
Sphincter preservation	74 (76)	Recurrence	85 (88)
Lymph node yield	72 (74)	Lymph node yield	83 (86)
Survival	71 (73)	Length of hospital stay	83 (86)

^a^High importance is defined as scoring 7–9 on a nine-point Likert scale.

The response rate in Round 2 was 80% (78/98) for health professionals and 90% (87/97) for patients. The provision of feedback and more stringent cut-off criteria in Round 2 resulted in 23 domains being retained for consideration in the consensus meetings (S2 Table).

### Phase 3: Consensus Meetings

The two professional and one patient/caregiver consensus meetings were attended by 61, 35, and 14 participants, respectively. Professional demographic details were not completed as planned and are therefore missing. At the Tripartite colorectal conference, anonymized voting did not reach a consensus on domains for the core set in the allotted time. Eight domains were voted “Out” and were discarded. The remainder were considered polarized with support for inclusion of between approximately 40% and 60% (Table 3), and these were brought forward to the European Society of Coloproctology meeting. Initial voting in this second meeting identified four domains to be included into the core set, five to be discarded, and six to be discussed further. Follow-up voting reached a consensus on including an additional two domains (Table 3). The composition of the final health professional core set of outcomes was ratified by a two-thirds majority.

**Table 3:** Voting on outcome domains to be included in the COS in the surgeon consensus meetings.

	Tripartite voting *N* = 61 n (%)*		ESCP^a^ Round 1 voting *N* = 35 n (%)*			ESCP^a^ Round 2 voting *N* = 35 n (%)*		
Outcome domain	In	Consensus	In	Out	Consensus	In	Out	Consensus
Anastomotic leak	36 (59)	Vote again	23 (65)	7 (20)	Vote again	20 (57)	14 (40)	In
Surgical site infection	29 (48)	Vote again	12 (35)	12 (35)	Vote again	8 (23)	27 (77)	Out
Hemorrhage	13 (21)	Out	-	-	-	-	-	-
Visceral injury	9 (14)	Out	-	-	-	-	-	-
Conversion to open operation	15 (24)	Out	-	-	-	-	-	-
Venous thromboembolism	18 (29)	Vote again	4 (10)	28 (80)	Out	-	-	-
Bowel obstruction	16 (26)	Vote again	7 (21)	23 (67)	Out	-	-	-
Abandoning the operation	3 (5)	Out	-	-	-	-	-	-
Perioperative mortality	38 (62)	Vote again	33 (95)	2 (5)	In	-	-	-
Reoperation	43 (71)	Vote again	19 (55)	6 (17)	Vote again	13 (38)	20 (56)	Out
Stoma rate	25 (41)	Vote again	14 (40)	15 (42)	Vote again	16 (45)	17 (48)	Out
Stoma complications	16 (26)	Vote again	8 (23)	24 (68)	Out	-	-	-
Readmission to hospital	21 (34)	Vote again	15 (44)	18 (51)	Vote again	8 (23)	30 (77)	Out
Cancer recurrence	27 (45)	Vote again	32 (90)	2 (5)	In	-	-	-
Long-term survival	31 (50)	Vote again	28 (80)	5 (15)	In	-	-	-
Resection margins	20 (33)	Vote again	27 (78)	3 (9)	In	-	-	-
Lymph node yield	10 (16)	Out	-	-	-	-	-	-
Length of stay in hospital	13 (22)	Out	-	-	-	-	-	-
Quality of life	34 (56)	Vote again	19 (53)	9 (27)	Vote again	25 (71)	9 (26)	In
Sexual functioning	21 (34)	Vote again	7 (20)	21 (60)	Out	-	-	-
Physical functioning	12 (19)	Out	-	-	-	-	-	-
Fecal urgency	12 (19)	Out	-	-	-	-	-	-
Fecal incontinence	27 (44)	Vote again	9 (25)	18 (50)	Out	-	-	-

^a^ European Society of Coloproctology

In initial anonymized voting at the patient consensus meeting, ten domains were voted “In,” four “Out,” and nine considered for further debate (Table 4). Extensive discussion ensued, and it was recognized that some domains had overlapping content and meaning. “Physical function” was therefore grouped with “quality of life,” and “resection margins” was grouped with “survival.” Follow-up voting reached a consensus on including three more domains into the core set. Patient and professional COSs were then combined (Box 1). Discussions around perioperative mortality were interesting. Patients were aware that CRC surgery typically has a low operative mortality and did not feel it important to differentiate between early mortality and survival in the context of identifying the minimum set of core outcomes. It was excluded after two rounds of voting. Surgeons, conversely, felt perioperative mortality was an important marker of surgical (technical) success, and it was voted into the COS.

**Table 4:** Voting on outcome domains to be included in the COS in the patient consensus meeting.

		Round 1 voting n (%)^a^				Round 2 voting n (%)^a^		
Outcome domain	In	Out	Unsure	Consensus	In	Out	Unsure	Consensus
Anastomotic leak	9 (64)	2 (14)	3 (21)	Vote again	11 (79)	2 (14)	1 (7)	In
Surgical site infection	14 (100)	0	0	In	-	-	-	-
Hemorrhage	7 (50)	2 (14)	5 (36)	Vote again	1 (7)	10 (71)	3 (21)	Out
Visceral injury	8 (57)	3 (21)	3 (21)	Vote again	4 (29)	10 (71)	0	Out
Conversion to open operation	12 (86)	1 (7)	1 (7)	In	-	-	-	-
Venous thromboembolism	7 (50)	4 (29)	3 (21)	Vote again	4 (29)	8 (57)	2 (14)	Out
Bowel obstruction	5 (36)	5 (36)	4 (29)	Vote again	2 (14)	11 (79)	1 (7)	Out
Abandoning the operation	4 (29)	7 (50)	3 (21)	Out	-	-	-	-
Perioperative mortality	7 (50)	5 (36)	2 (14)	Vote again	4 (29)	10 (71)	0	Out
Reoperation	3 (21)	8 (57)	3 (21)	Out	-	-	-	-
Stoma rate	13 (93)	1 (7)	0	In	-	-	-	-
Stoma complications	6 (43)	6 (43)	2 (14)	Vote again	13 (93)	1 (7)	0	In
Readmission to hospital	5 (36)	8 (57)	1 (7)	Out	-	-	-	-
Cancer recurrence	9 (64)	4 (29)	1 (7)	In	-	-	-	-
Long-term survival	10 (71)	4 (29)	0	In	-	-	-	-
Resection margins	11 (79)	1 (7)	2 (14)	In	-	-	-	-
Lymph node yield	7 (50)	6 (43)	1 (7)	Vote again	0	13 (93)	1 (7)	Out
Length of stay in hospital	0	14 (100)	1 (7)	Out	-	-	-	-
Quality of life	12 (86)	1 (7)	1 (7)	In	-	-	-	-
Sexual functioning	7 (50)	3 (21)	4 (29)	Vote again	11 (79)	3 (21)	0	In
Physical functioning	10 (71)	4 (29)	0	In	-	-	-	-
Fecal urgency	10 (71)	1 (7)	3 (21)	In	-	-	-	-
Fecal incontinence	10 (71)	2 (14)	2 (14)	In	-	-	-	-

^**a**^ Patients voted for whether each domain was in or out of the COS.

**Box 1. Final COS**

**Oncological outcomes:**
- Long-term survival
- Cancer recurrence
- Resection margins

**Operative outcomes:**
- Anastomotic leak
- Perioperative survival
- Surgical site infection
- Stoma rates and complications
- Conversion to open operation (where appropriate)

**Quality of life:**
- Physical function
- Sexual function
- Fecal incontinence
- Fecal urgency

## Conclusions

This study has determined a COS to use in trials in CRC surgery. A wide range of sources including published studies and patient interviews were used to identify the initial long list that was reduced using consensus methods with professionals and patients to identify 23 domains of the greatest importance. Finally, consensus meetings with surgeons and patients and caregivers reconsidered the domains and voted on the final COS. It is recommended that all trials and other nonrandomized studies and audit undertaking of clinical evaluation of CRC surgery use this COS and further work to establish best instruments with which to measure these outcomes is underway.

It was not possible to identify other published COSs for CRC surgery. The COMET database has no other CRC COS development projects registered, although there is ongoing research to define a COS for anal cancer trials that may be conceptually similar [21]. A COS for use in all types of adult cancer treatment trials has been developed [22]. This generic cancer COS was developed with face-to-face consensus meetings with professionals who recommended that 12 symptoms be included in a COS (fatigue, insomnia, pain, anorexia, dyspnea, cognitive problems, anxiety, nausea, depression, sensory neuropathy, constipation, and diarrhea). It did not, however, survey patients’ views, which are very important in the evaluation of treatments [23]. Conceptually, it has been argued that patients’ views should be given at least equal if not greater importance over those of health professionals [24], and it is therefore unclear if this represents an appropriate COS. Furthermore, the scope of this COS encompasses all cancer treatments in adults. This broad remit may neglect details that are of specific importance to CRC patients or indeed patients undergoing surgery.

This study used robust consensus methodology and followed guidelines established by the COMET initiative to develop a COS, but there are some weaknesses. In Phase 1, the identification of large numbers of outcomes from primary data sources mandated the categorization into domains. This introduces an element of subjectivity that was minimized through independent dual categorization, although there is the possibility that some outcomes may have been inappropriately grouped or separated. This is highlighted by the additional amalgamation of domains that occurred during the consensus meetings, where participants considered some domains unnecessarily detailed. In Phase 2, the scope of the Delphi process was limited to the UK before the COS development process was opened internationally to professionals in Phase 3. This was done to exclude the least important domains without the complexity of a multinational Delphi process; however, different domains may have been brought forward for discussion at the consensus meetings if the first round had included international participation. Participants at the professional consensus meeting did not report their country of residence as planned. This was not apparent until after the meeting had concluded, and it is therefore unclear as to the precise nationalities involved in the process. Further research is therefore needed to fully validate the COS more widely. This will include liaising with international organizations including the European Organisation for Research and Treatment of Cancer and the United States National Cancer Institute.

Another limitation is the numbers of participants involved in the process and response rates. In particularly, the response rates from patients to the first questionnaire survey was low. The effect of this on the validity of the Delphi is unclear, because the purpose of the methodology is not to garner the views of a representative sample of stakeholders but to gain a consensus among a wide range of individuals with disparate opinions. In that respect, this study achieved wide diversity based on a priori patient characteristics. However, it is possible that patients not responding to the survey may have different opinions of the importance of each item to the responding group. Similarly, different professional groups, such as medical oncologists and radiologists, could have been recruited to bring a different perspective to the COS. The scope of the COS was, however, limited to surgery, and this guided the stakeholder involvement. It is important to expand the COS to include all treatment modalities in the future, at which point the involvement of other groups will be critical.

The scope of this COS was intentionally broad and included cancer of the colon and rectum. Many of the COS domains clearly traverse all colorectal surgery and include oncological outcomes such as survival, surgical outcomes such as anastomotic leak, and quality of life outcomes such as physical function. It is acknowledged, however, that patients have different experiences following surgery for colon or rectal cancer. Problems with sexual or bowel function, for example, are typically caused by the pelvic dissection and loss of reservoir associated with rectal surgery and are not usually associated with right-sided colonic surgery. Similarly, stoma formation is rare following right hemicolectomy. This issue was discussed at length in the professional consensus meetings and, although most participants agreed on a combined colorectal COS, some professionals still considered it unresolved. Nonetheless, feedback from patients suggested that these outcomes were important to measure in all colorectal studies, because the information was valued. In that respect, a patient undergoing right hemicolectomy may be concerned about the need for a stoma but reassured by a body of research demonstrating that stoma rates are low. Ultimately, the decision to have a combined colorectal COS was based on a patient-centered approach.

This study has defined which outcomes to measure in studies of CRC surgery. The next step is to identify how these outcomes should be measured in a valid, reliable, and acceptable way. This was not considered within this study because it is first necessary to assess the quality of potential outcome measures, a process that could not be undertaken until the COS domains were defined. One organization championing standards in measurement instruments is COSMIN (COnsensus-based Standards for the selection of health Measurement Instruments) [25]. This group uses similar Delphi methods to agree on the taxonomy, terminology, and definition of outcomes—a process that will be necessary to further the benefits of this COS. Another potential benefit of COSs is to provide evidence for use in clinical discussions with patients. Future research is required to examine how the COS can be included in clinical consultations to inform patient-centered decision making.

In conclusion, this study used health services research methodology to develop a COS for use in CRC surgical trials. It is now necessary to validate the use of this set in international research practice, with the aim of maximizing cross study comparisons, easing meta-analysis, and minimizing outcome reporting bias. Further work to identify recommended measures to use to assess each outcome is underway.

## Supporting Information

S1 Table Patient and professional scoring of outcome domains in Round 1, including details of which domains were retained by patients, professionals, and overall. Domains were retained if rated of high importance by over 50% of respondents and low importance by less than 15% of respondents. Domains were retained overall if they were retained by either stakeholder.
(DOCX)

S2 Table Patient and professional scoring of outcome domains in Round 2, including details of which domains were retained by patients, professionals, and overall. Domains were retained if rated of high importance by over 70% of respondents and low importance by less than 15% of respondents. Domains were retained overall if they were retained by either stakeholder.
(DOCX)

## Abbreviations

COS: core outcome set
COSMIN: Consensus-based Standards for the selection of health Measurement Instruments
COMET: Core Outcome Measures in Effectiveness Trials
CRC: colorectal cancer
RCT: randomized controlled trial
