# Supplementary material for: Core Outcomes for Colorectal Cancer Surgery: A Consensus Study
Source: PLoS Med. 2016 Aug 9;13(8):e1002071. doi: 10.1371/journal.pmed.1002071 (PMC4978448; doi:10.1371/journal.pmed.1002071)
Supplement: S1 Table — Domains were retained if rated of high importance by over 50% of respondents and low importance by less than 15% of respondents. Domains were retained overall if they were retained by either stakeholder. (DOCX) [file pmed.1002071.s001.docx]

| Outcome domain | n (%) patients rating domain high importance^a^ | n (%) patients rating domain low importance^b^ | Domain retained by patients^c^ | n (%) professionals rating domain high importance^a^ | n (%) professionals rating domain low importance^b^ | Domain retained by professionals^c^ | Domain retained overall^d^ |
| --- | --- | --- | --- | --- | --- | --- | --- |
| Stoma rate | 84(87) | 3(3) | Yes | 82(84) | 0(0) | Yes | Yes |
| Problems with stomata | 57(59) | 20(21) | No | 53(55) | 13(13) | Yes | Yes |
| Anastomotic leak | 63(65) | 16(16) | No | 96(99) | 0(0) | Yes | Yes |
| Surgical site infection | 60(62) | 18(19) | No | 72(74) | 4(4) | Yes | Yes |
| Bowel obstruction | 56(58) | 9(9) | Yes | 44(44) | 14(14) | No | Yes |
| Pneumonia | 36(37) | 27(28) | No | 53(55) | 17(17) | Yes | Yes |
| Myocardial infarction | 40(41) | 27(28) | No | 54(56) | 14(14) | Yes | Yes |
| Venous thromboembolism | 45(46) | 15(15) | No | 73(75) | 6(6) | Yes | Yes |
| Operative blood loss | 30(31) | 33(34) | No | 54(56) | 14(14) | Yes | Yes |
| postoperative haemorrhage | 36(37) | 31(32) | No | 73(75) | 9(9) | Yes | Yes |
| Conversion to open operation | 53(55) | 14(14) | Yes | 88(91) | 1(1) | Yes | Yes |
| Visceral injury | 47(48) | 20(21) | No | 73(75) | 5(5) | Yes | Yes |
| Lymph node yield | 72(74) | 14(14) | Yes | 83(86) | 8(8) | Yes | Yes |
| Resection margins | 88(91) | 2(2) | Yes | 93(96) | 2(2) | Yes | Yes |
| Non-progression | 80(82) | 10(10) | Yes | 77(79) | 6(6) | Yes | Yes |
| Length of time after surgery to start eating and drinking | 50(52) | 17(18) | No | 53(55) | 8(8) | Yes | Yes |
| Length of time after surgery until the bowels open | 56(58) | 9(9) | Yes | 41(41) | 11(11) | No | Yes |
| Length of hospital stay | 49(51) | 14(14) | Yes | 83(86) | 4(4) | Yes | Yes |
| Unplanned readmission | 30(31) | 31(32) | No | 81(83) | 5(5) | Yes | Yes |
| Reoperation | 42(43) | 24(25) | No | 87(90) | 4(4) | Yes | Yes |
| Operative mortality | 43(44) | 34(35) | No | 89(92) | 2(2) | Yes | Yes |
| Survival | 71(73) | 12(12) | Yes | 76(78) | 6(6) | Yes | Yes |
| Recurrence | 80(82) | 7(7) | Yes | 85(88) | 4(4) | Yes | Yes |
| Local recurrence | 80(82) | 6(6) | Yes | 86(89) | 5(5) | Yes | Yes |
| Distant recurrence | 81(83) | 8(8) | Yes | 87(90) | 5(5) | Yes | Yes |
| Disease-free interval | 79(81) | 10(10) | Yes | 72(74) | 8(8) | Yes | Yes |
| General pain | 61(63) | 11(11) | Yes | 56(58) | 13(13) | Yes | Yes |
| Diarrhoea | 52(54) | 6(6) | Yes | 44(44) | 19(19) | No | Yes |
| Constipation | 51(53) | 8(8) | Yes | 30(30) | 32(32) | No | Yes |
| Faecal frequency | 46(47) | 8(8) | No | 52(54) | 12(12) | Yes | Yes |
| Faecal urgency | 51(53) | 8(8) | Yes | 61(63) | 10(10) | Yes | Yes |
| Faecal discrimination | 50(52) | 9(9) | Yes | 44(44) | 17(17) | No | Yes |
| Faecal incontinence | 59(61) | 7(7) | Yes | 63(65) | 10(10) | Yes | Yes |
| Rectal bleeding | 62(64) | 9(9) | Yes | 38(38) | 30(30) | No | Yes |
| Problems with the stoma | 66(68) | 6(6) | Yes | 42(42) | 29(29) | No | Yes |
| The need for extra aids to control symptoms, for example pain killers or incontinence pads | 56(58) | 14(14) | Yes | 45(45) | 24(24) | No | Yes |
| Physical function | 70(72) | 6(6) | Yes | 40(40) | 18(18) | No | Yes |
| Self-care | 62(64) | 4(4) | Yes | 48(48) | 19(19) | No | Yes |
| Role function | 64(66) | 6(6) | Yes | 49(51) | 17(17) | No | Yes |
| Cognition | 50(52) | 14(14) | Yes | 23(23) | 42(42) | No | Yes |
| Overall health | 59(61) | 3(3) | Yes | 50(52) | 22(22) | No | Yes |
| Overall quality of life | 64(66) | 7(7) | Yes | 69(71) | 10(10) | Yes | Yes |
| Ability to cope emotionally | 63(65) | 9(9) | Yes | 37(37) | 30(30) | No | Yes |
| Outlook on life | 63(65) | 11(11) | Yes | 37(37) | 33(33) | No | Yes |
| Sphincter preservation* | 74 (76) | 14(14) | Yes | - | - | - | - |
| Sexual function | 47(48) | 17(18) | No | 80(82) | 5(5) | Yes | Yes |
| Wound dehiscence | 50(52) | 21(22) | No | 44(44) | 20(20) | No | No |
| Wound seroma | 39(40) | 27(28) | No | 10(10) | 47(47) | No | No |
| Delayed wound healing | 45(46) | 21(22) | No | 34(34) | 25(25) | No | No |
| Hernia | 46(47) | 14(14) | No | 35(35) | 21(21) | No | No |
| Ileus | 47(48) | 17(18) | No | 34(34) | 25(25) | No | No |
| Enterovisceral fistula | 47(48) | 20(21) | No | 48(48) | 28(28) | No | No |
| Enterocutaneous fistula | 50(52) | 19(20) | No | 50(52) | 23(23) | No | No |
| Ascites | 51(53) | 19(20) | No | 29(29) | 45(45) | No | No |
| Urinary tract infection | 35(36) | 30(31) | No | 21(21) | 41(41) | No | No |
| Renal failure | 44(45) | 31(32) | No | 36(36) | 29(29) | No | No |
| Non-infective respiratory complications | 33(34) | 32(33) | No | 24(24) | 29(29) | No | No |
| Arrhythmia | 38(39) | 31(32) | No | 32(32) | 28(28) | No | No |
| Non-ischaemic/non-arrhythmic cardiac complications | 38(39) | 29(30) | No | 30(30) | 32(32) | No | No |
| Cerebrovascular complications | 38(39) | 25(26) | No | 40(40) | 34(34) | No | No |
| Septicaemia | 37(38) | 30(31) | No | 49(49) | 21(21) | No | No |
| Thrombophlebitis | 31(32) | 35(36) | No | 21(21) | 44(44) | No | No |
| Rash or other skin problems | 22(23) | 38(39) | No | 10(10) | 69(71) | No | No |
| Allergic reactions | 29(30) | 29(30) | No | 34(34) | 48(48) | No | No |
| Multi-organ failure | 39(40) | 30(31) | No | 61(63) | 17(17) | No | No |
| Catheter complications | 29(30) | 35(36) | No | 14(14) | 51(53) | No | No |
| Incision size | 42(43) | 26(27) | No | 38(38) | 27(27) | No | No |
| Operative time | 43(44) | 18(19) | No | 45(45) | 16(16) | No | No |
| Equipment failure | 21(22) | 43(44) | No | 35(35) | 43(43) | No | No |
| Length of bowel removed | 54(56) | 15(15) | No | 35(35) | 35(35) | No | No |
| Fatigue | 50(52) | 19(20) | No | 24(24) | 34(34) | No | No |
| Appearance/weight loss | 43(44) | 26(27) | No | 27(27) | 33(33) | No | No |
| Feeling faint | 35(36) | 24(25) | No | 7(7) | 64(66) | No | No |
| Insomnia | 34(35) | 20(21) | No | 11(11) | 59(61) | No | No |
| Shortness of breath | 43(44) | 18(19) | No | 18(18) | 49(49) | No | No |
| Anorexia | 35(36) | 24(25) | No | 15(15) | 43(43) | No | No |
| Dysphagia/regurgitation | 42(43) | 19(20) | No | 12(12) | 56(58) | No | No |
| Reflux | 39(40) | 21(22) | No | 11(11) | 58(60) | No | No |
| Belching, gas or flatulence | 39(40) | 18(19) | No | 7(7) | 68(70) | No | No |
| Nausia and vomiting | 44(45) | 11(11) | No | 26(26) | 31(31) | No | No |
| Tenesmus | 46(47) | 16(16) | No | 37(37) | 22(22) | No | No |
| How long it takes for a bowel movement | 37(38) | 19(20) | No | 17(17) | 51(53) | No | No |
| Bloating | 42(43) | 12(12) | No | 11(11) | 55(57) | No | No |
| Urinary frequency | 43(44) | 14(14) | No | 19(19) | 49(51) | No | No |
| Urinary incontinence | 44(45) | 15(15) | No | 33(33) | 38(38) | No | No |
| Dysuria | 42(43) | 14(14) | No | 14(14) | 57(59) | No | No |
| Problems with periods (female) | 27(28) | 9(9) | No | 17(17) | 50(52) | No | No |
| Social function | 38(39) | 17(18) | No | 17(17) | 51(53) | No | No |
| Spiritual or faith issues | 13(13) | 46(47) | No | 9(9) | 66(68) | No | No |
| Finances | 40(41) | 32(33) | No | 21(21) | 59(61) | No | No |
| Emotions - Anxiety | 42(43) | 23(24) | No | 35(35) | 32(32) | No | No |
| Emotions – Depression | 45(46) | 25(26) | No | 35(35) | 32(32) | No | No |

^a^High importance is defined as scoring 7-9 on a nine-point Likert scale

^b^Low importance is defined as scoring 1-3 on a nine-point Likert scale

^c^Domain retained if rated between 7-9 by over 50% of respondents and between 1-3 by less than 15%.

^d^Domain retained for Round 2 if it was retained by either patients or professionals.

*Patient feedback resulted in the combination of domains “Sphincter preservation” into “Stoma rates” in Round 1, as these concepts were considered synonymous. “Sphincter preservation” was therefore not included in the Round 1 professional questionnaire.
