# Supplementary material for: Core Outcomes for Colorectal Cancer Surgery: A Consensus Study
Source: PLoS Med. 2016 Aug 9;13(8):e1002071. doi: 10.1371/journal.pmed.1002071 (PMC4978448; doi:10.1371/journal.pmed.1002071)
Supplement: S2 Table — Domains were retained if rated of high importance by over 70% of respondents and low importance by less than 15% of respondents. Domains were retained overall if they were retained by either stakeholder. (DOCX) [file pmed.1002071.s002.docx]

| Outcome domain | n (%) patients rating domain high importance^a^ | n (%) patients rating domain low importance^b^ | Domain retained by patients^c^ | n (%) professionals rating domain high importance^a^ | n (%) professionals rating domain low importance^b^ | Domain retained by professionals^c^ | Domain retained overall^d^ |
| --- | --- | --- | --- | --- | --- | --- | --- |
| Stoma rate | 82(94) | 1(1) | Yes | 76(98) | 0(0) | Yes | Yes |
| Anastomotic leak | 67(77) | 5(6) | Yes | 78(100) | 0(0) | Yes | Yes |
| Surgical site infection | 61(70) | 6(7) | Yes | 70(90) | 0(0) | Yes | Yes |
| Bowel obstruction | 62(71) | 2(2) | Yes | 39(50) | 4(5) | No | Yes |
| postoperative haemorrhage | 32(37) | 17(20) | No | 55(70) | 4(5) | Yes | Yes |
| Conversion to open operation | 48(55) | 8(9) | No | 72(93) | 0(0) | Yes | Yes |
| Visceral injury | 48(55) | 4(5) | No | 68(88) | 0(0) | Yes | Yes |
| Lymph node harvest | 55(63) | 3(3) | No | 64(83) | 2(3) | Yes | Yes |
| Resection margins | 82(94) | 1(1) | Yes | 78(100) | 0(0) | Yes | Yes |
| Non-progression | 69(79) | 2(2) | Yes | 72(93) | 2(3) | Yes | Yes |
| Length of hospital stay | 39(45) | 7(8) | No | 62(80) | 0(0) | Yes | Yes |
| Unplanned readmission | 16(18) | 13(15) | No | 70(90) | 2(3) | Yes | Yes |
| Reoperation | 33(38) | 11(13) | No | 72(93) | 0(0) | Yes | Yes |
| Operative mortality | 37(43) | 13(15) | No | 76(98) | 0(0) | Yes | Yes |
| Survival | 61(70) | 6(7) | Yes | 68(88) | 2(3) | Yes | Yes |
| Recurrence | 72(83) | 1(1) | Yes | 74(95) | 2(3) | Yes | Yes |
| Local recurrence | 73(84) | 2(2) | Yes | 74(95) | 2(3) | Yes | Yes |
| Distant recurrence | 74(85) | 2(2) | Yes | 76(98) | 2(3) | Yes | Yes |
| Disease-free interval | 67(77) | 4(5) | Yes | 72(93) | 2(3) | Yes | Yes |
| Problems with the stoma | 62(71) | 2(2) | Yes | 33(43) | 14(18) | No | Yes |
| Physical function | 62(71) | 2(2) | Yes | 29(38) | 8(10) | No | Yes |
| Overall quality of life | 62(71) | 4(5) | Yes | 55(70) | 4(5) | Yes | Yes |
| Sexual function | 48(55) | 7(8) | No | 59(75) | 4(5) | Yes | Yes |
| Problems with stomata | 57(66) | 10(11) | No | 35(45) | 6(8) | No | No |
| Pneumonia | 27(31) | 12(14) | No | 29(38) | 8(10) | No | No |
| Myocardial infarction | 30(34) | 12(14) | No | 31(40) | 6(8) | No | No |
| Venous thromboembolism | 45(52) | 4(5) | No | 53(68) | 6(8) | No | No |
| Operative blood loss | 22(25) | 20(23) | No | 43(55) | 4(5) | No | No |
| Length of time after surgery to start eating and drinking | 36(41) | 7(8) | No | 31(40) | 2(3) | No | No |
| Length of time after surgery until the bowels open | 50(57) | 4(5) | No | 35(45) | 4(5) | No | No |
| General pain | 54(62) | 7(8) | No | 39(50) | 16(20) | No | No |
| Diarrhoea | 46(53) | 5(6) | No | 39(50) | 14(18) | No | No |
| Constipation | 39(45) | 6(7) | No | 14(18) | 18(23) | No | No |
| Faecal frequency | 40(46) | 2(2) | No | 41(53) | 2(3) | No | No |
| Faecal urgency | 52(60) | 3(3) | No | 53(68) | 6(8) | No | No |
| Faecal discrimination | 41(47) | 4(5) | No | 31(40) | 10(13) | No | No |
| Faecal incontinence | 60(69) | 0(0) | No | 53(68) | 6(8) | No | No |
| Rectal bleeding | 57(66) | 3(3) | No | 29(38) | 16(20) | No | No |
| The need for extra aids to control symptoms, for example pain killers or incontinence pads | 47(54) | 4(5) | No | 27(35) | 16(20) | No | No |
| Self-care | 59(68) | 0(0) | No | 39(50) | 10(13) | No | No |
| Role function | 59(68) | 3(3) | No | 23(30) | 16(20) | No | No |
| Cognition | 34(39) | 10(11) | No | 18(23) | 21(28) | No | No |
| Overall health | 53(61) | 5(6) | No | 43(55) | 10(13) | No | No |
| Ability to cope emotionally | 58(67) | 8(9) | No | 33(43) | 14(18) | No | No |
| Outlook on life | 54(62) | 7(8) | No | 41(53) | 16(20) | No | No |

^a^High importance is defined as scoring 7-9 on a nine-point Likert scale

^b^Low importance is defined as scoring 1-3 on a nine-point Likert scale

^c^Domain retained if rated between 7-9 by over 70% of respondents and between 1-3 by less than 15%.

^d^Domain retained for the consensus meeting if it was retained by either patients or professionals.
